# Autism spectrum disorder early in development associated with *CHD8* mutations among two Chinese children

**DOI:** 10.1186/s12887-018-1307-4

**Published:** 2018-10-30

**Authors:** Jiangping Wang, Jinling Liu, Yi Gao, Kaixuan Wang, Kewen Jiang

**Affiliations:** 1 Department of Rehabilitation, The Children’s Hospital Zhejiang University School of Medicine, 3333 Binsheng Road, Hangzhou, 310051 China; 2 Department of Respiration, The Children’s Hospital Zhejiang University School of Medicine, 3333 Binsheng Road, Hangzhou, 310051 China; 3 Department of Neurology, The Children’s Hospital Zhejiang University School of Medicine, 3333 Binsheng Road, Hangzhou, 310051 China; 4 Department of Pediatrics, Jinhua Central Hospital, Jinhua, 321000 Zhejiang Province China; 5 Department of Laboratory, The Children’s Hospital Zhejiang University School of Medicine, 3333 Binsheng Road, Hangzhou, 310051 China

**Keywords:** Autism spectrum disorders, Chromodomain helicase DNA-binding protein 8, Next-generation sequencing

## Abstract

**Background:**

Autism spectrum disorder (ASD) is a heterogeneous group of neurodevelopmental disorders. Genetically based subtype identification may prove more beneficial not only in illuminating the course and prognosis, but also for individualized treatment targets of an ASD sub-group. Increasing evidence has shown that de novo loss-of-function mutations in the chromodomain helicase DNA-binding protein 8 (*CHD8*) gene are associated with an ASD sub-group.

**Case presentation:**

Here we describe two ASD cases in children with mild intellectual disability, early motor deficits, and speech delay, without distinct structural or EEG brain anomalies. Exome sequencing revealed a novel heterozygous nonsense/missense mutations(c.2647C > A/p.E883X and c.1677C > A/p.M559I respectively) in *CHD8* gene.

**Conclusions:**

There were few cases in the literature reporting de novo mutation of *CHD8* in ASD. As demonstrated in our patients, along with other previously reported studies support that disruption of the *CHD8* gene represents a specific genetic sub-type of ASD.

## Background

Autism spectrum disorder (ASD) is a heterogeneous group of neurodevelopmental conditions with significant genotypic complexity usually diagnosed in early childhood that are characterized by impairments in communication, social interaction, and by repetitive patterns of behavior [1, 2]. There may be as many as 10 to 20 million people affected by ASD in China [3]. However, the cause of ASD remains unknown in approximately 80% of patients [4]. Although more than 100 genes and genomic regions have been associated with ASD [5], and > 800 genes have been suggested to play a role in ASD [6–9], these have not been tied to the ASD’s complicate sub-type phenotypes. Increasing evidences suggesting that genetically based subtype identification may prove more beneficial not only in illuminating the course and prognosis of a sub-group of individuals with ASD, but also for individualized treatment targets [10].

The chromodomain helicase DNA binding protein 8 (*CHD8*) on 14q11.2 has been reported to be associated with ASD. It has been reported with a variety of genotypes including chromosomal microdeletions [11], balanced chromosomal abnormalities [12], haploinsufficiency of the gene due to a 2.89 Mb deletion [11], and a recurrent ~ 100 Kb microdeletion [13]. In addition to cytogenetic studies, next-generation sequencing (NGS) technologies performed in ASD cohorts have discovered loci associated with an increased risk of ASD [7–9]. Increasing evidence has indicated that de novo loss of function mutations contribute to ASD risk [14–16]. It has been reported that *CHD8* mutations could result in a behavioral profile consistent with ASD, together with macrocephaly, distinct facial features, and gastrointestinal complaints [17]. In this paper, we report two cases of children with ASD and global developmental delays diagnosed based on the clinical findings and confirmed by genetic tests with a de novo mutation of *CHD8* that has not been previously reported.

## Case presentation

### Case 1

**Fig. 1:**
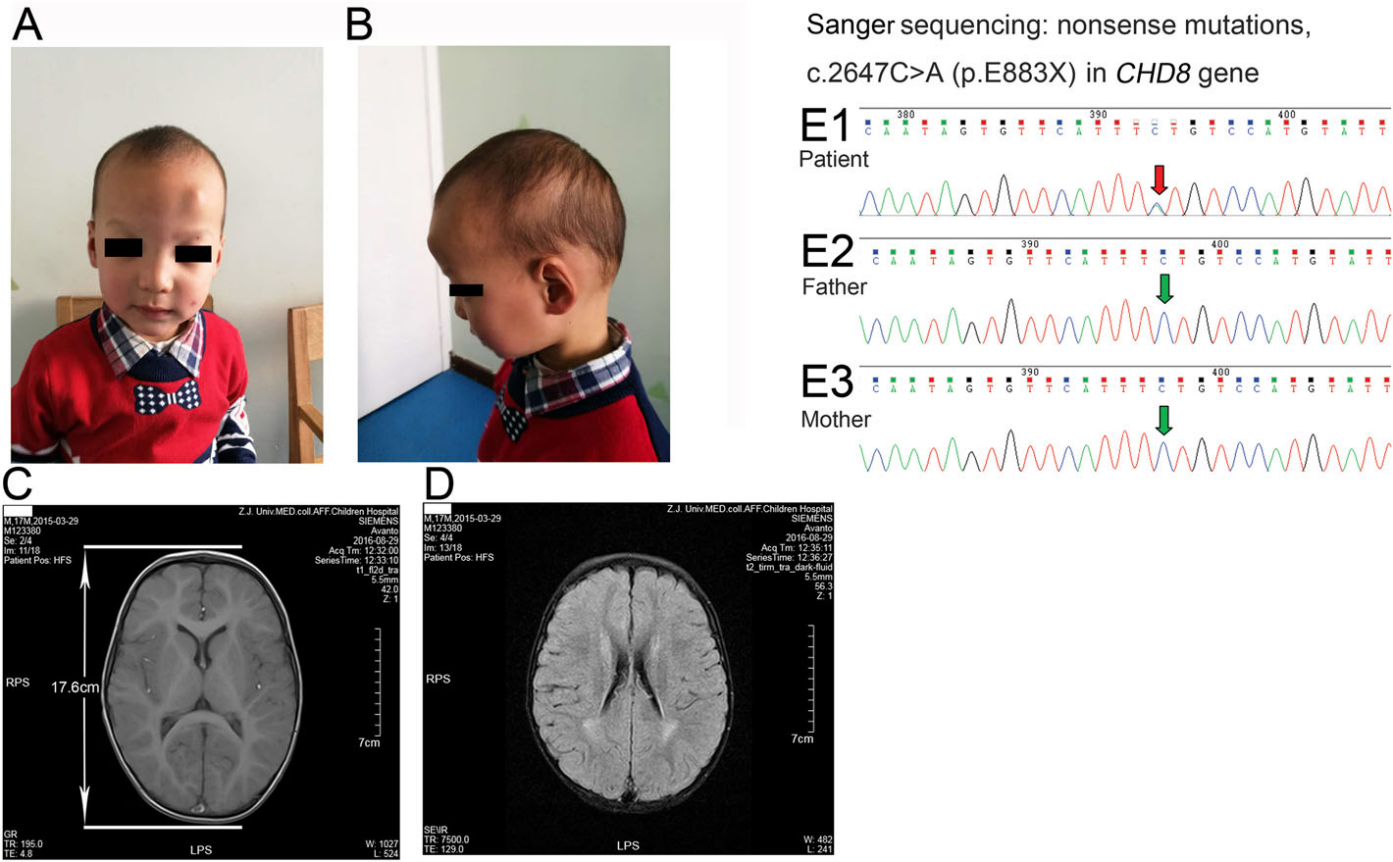
Dysmorphic features, brain MRI and exome sequencing of patient 1. **a**: Anterior photograph of the proband at 24-months of age. **b**: Lateral photograph of the proband at 24-months of age. **c** MRI showed the enlargement of skull anteroposterior diameter (17.6 cm). **d** MRI showed Increased signal in T2 in the white matter territories adjacent to the lateral ventricles and subcortical zone, and retardation of brain development. E1: Exome sequencing revealed a novel heterozygous nonsense mutations, c.2647C > A (p.E883X) in *CHD8* gene which was further validated by Sanger sequencing, but not seen in his parents E2 and E3

The 24-month-old boy was the first child of healthy nonconsanguineous parents. Pregnancy and delivery were normal. He was born at term with normal measurements (birth weight: 3550 g, 50-85th percentile). The physical development seemed over growth of his infanthood, as his weight, length and head circumference was 5200 g (> 85th percentile), 59.5 cm (> 97th percentile) and 40 cm (> 97th percentile) separately at 1-month old, as well as 11.6 kg (85-97th percentile), 86 cm (> 97th percentile) and 47.5 cm (> 85th percentile) separately at 1-year old. He was irritable when kept in the crawling position at 3–4 months old presenting with crying constantly. He was neither to respond to his name nor to learn to talk since then. He presented with a general developmental delay and dysmorphic feature (Fig. 1a and b). He started sitting at 7-months old and walking at 15-months old but had never walked on all fours. He was always found tumbling head over heels without awareness of self protection. General learning difficulties were also observed. Subsequently, there were concerns about his delayed language development and abnormal behavior. He also showed symptoms of diarrhea and constipation alternately. Half month to 20 days with a dilute stool (3–4 stools/day), alternately turn to constipation (one stool/2–3 days) after medication, without anal fissure. He further had a halitosis in the morning due to the gastroesophageal reflux. He had an initial developmental evaluation at 17-months old with a subsequent follow-up. His hearing evaluation at 20-months of age was normal. He was not found with funicular hydrocele on the right until 12-months old and received repairing operation at 20-months old. There was no similar disease in the other member of the family. In the present study, the Bayley scales was chosen as an instrument to assess his neuropsychological profiles. The scales is an individually administered instrument, which assesses the cognitive, language, and motor functioning of infants and young children aged 1–42 months. The patient was assessed at 26 months old and the cognitive score was < 50 (18-19 months) and the motor score was 56 (19 months).

We used Peabody developmental motor scales 2nd edition (PDMS-2) to assessed the patient’s motor development which including subtest scores: balance ability (19 months), locomotion (18 months), grasping (20 months), visual-motor (V-M) integration (19 months), and standardized motor quotients: gross (GMQ) (74), fine (FMQ) (82), total motor(TMQ) (75).

Autism Diagnostic Observation Schedule (ADOS) [18] was used to evaluate communication, social interaction abilities, creativity and the imaginative use of objects (Table 1). In the “communication” category, it was noted that he did not respond to his name, retrieve objects, initiate interactions, imitation, or point to pictures or objects. He did not differentially respond to his mother’s voice from others. He still had not developed speech appropriately. We also observed poor and limited eye contact in reciprocal social interactions during the ADOS examination. He had abnormal social interactions with repetitive patterns of behavior, poor eye contacts and restricted interests. In terms of behavior, he was hyperactive with difficulty in sustaining attention on using specific objects, and was unable to follow one-step directions. He did not respond or react to the examiner’s emotional state (ADOS Social interaction score: 13; Autism Cut-Off: 7/ASD Cut-Off: 4) [18]. Concerning imagination, he did not initiate any spontaneous creative actions or pretend plays, even when he was invited to do so. His behavior did not reveal any unusual sensory interests, but the examiner noted the presentation with hyperactive behavior in sustaining attention on using specific objects and not to follow one-step directions. He also chewed on and ate non-edible objects. He showed little awareness of potential dangers. These findings confirmed our primary developmental diagnosis of ASD, which has finally been aligned to the American Psychiatric Association’s Diagnostic and Statistical Manual for Mental Disorders, 5th Edition (DSM5) criteria.

**Table 1:** Autism diagnostic observation schedule scores in Patients 1 and 2

	Patient 1	Patient 2
ADOS (module 4)		
Age at evaluation (months)	24	28
Communication (cut-off autism = 4, ASD = 2)	2	4
Social interaction (cut-off autism = 7, ASD = 4)	13	6
Total communication + social (cut-off autism = 12, ASD = 7)	15	10
Imagination	5	2
Restricted behaviors and interests	2	4
ADOS diagnosis	Autism	Autism
Final research diagnosis	Autism	Autism

MRI showed the enlargement of skull anteroposterior diameter (17.6 cm at 17-months, Fig. 1c), increased signal in T2 in the white matter territories adjacent to the lateral ventricles and subcortical zone, and retardation of brain development (Fig. 1d). EEG showed non specific slow background activity, as well as no epileptiform discharges.

Genetic analyses were performed after obtaining the patient’s signed informed consent and approved by our hospital ethical committee. Exome sequencing revealed a novel heterozygous nonsense mutations, c.2647C > A (p.E883X) in *CHD8* gene which was further validated by Sanger sequencing (Fig. 1. E1).

### Case 2

**Fig. 2:**
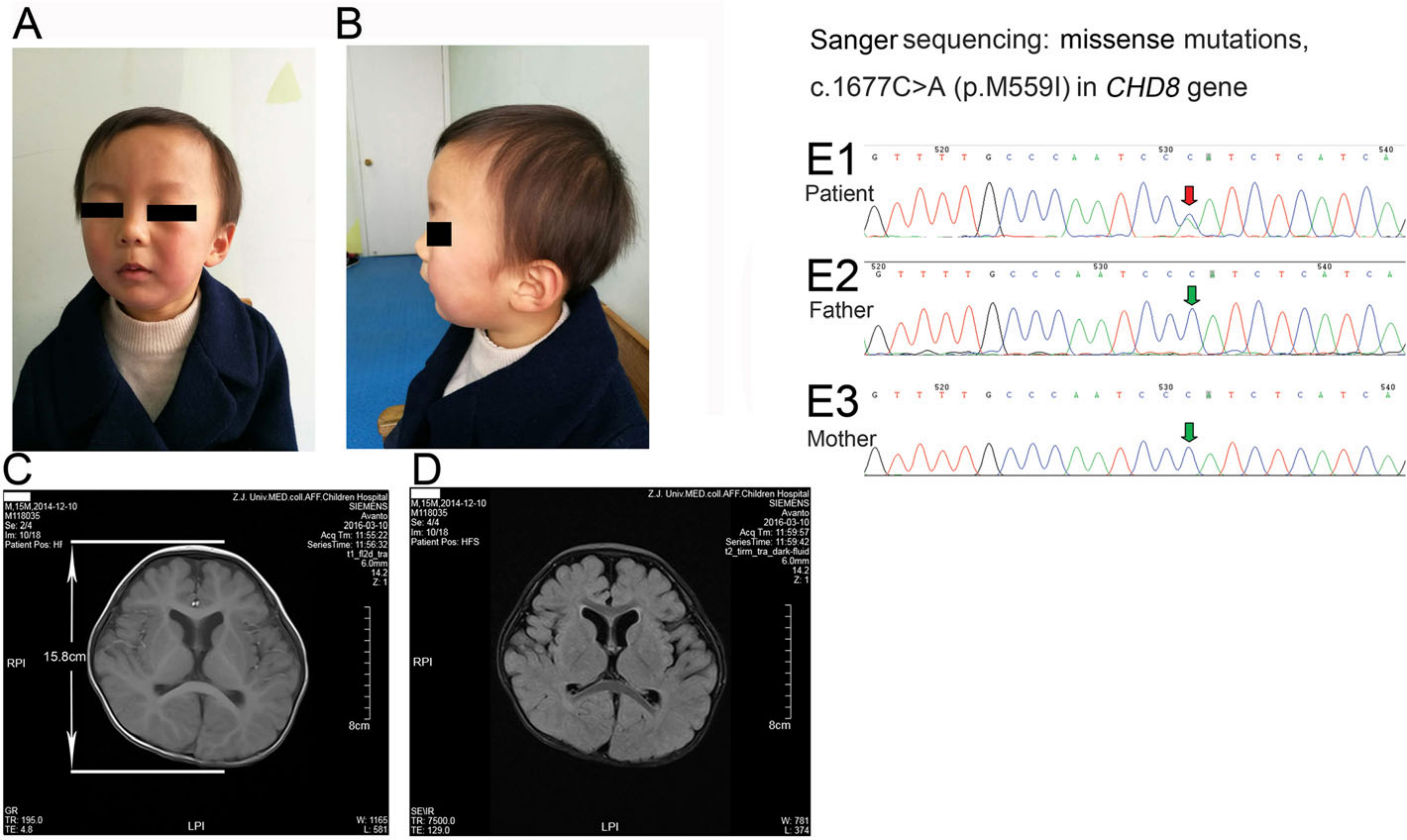
Dysmorphic features, brain MRI and exome sequencing of patient 2. **a** Anterior photograph of the proband at 24-months of age. **b** Lateral photograph of the proband at 24-months of age. **c** MRI showed the normal range of skull anteroposterior diameter. **d** MRI showed the increased signal in T2 in the white matter territories adjacent to the lateral ventricles and subcortical zone, and retardation of brain development. E1: Exome sequencing revealed a novel heterozygous missense mutations, c.1677C > A (p.M559I) in *CHD8* gene which was further validated by Sanger sequencing, but not seen in his parents E2 and E3

The proband is a 28-month-old boy who was born full term without major prenatal complications. The patient was the second child of healthy nonconsanguineous parents. His 5-year-old sister is healthy. Pregnancy and delivery were normal (birth weight: 3200 g, 50th percentile; length: 52 cm, 50-85th percentile). There were no major postnatal complications or congenital findings. The physical development also seemed over growth of his infanthood, as the weight, length and head circumference was 5200 g (50-85th percentile), 60 cm (97th percentile), and 40.4 cm (> 97th percentile) separately at 42-days old, as well as 17.5 kg (>97th percentile), 104 cm (>97th percentile), and 52 cm (> 97th percentile) separately at 2-years old. No facial or corporeal dysmorphic features have been detected (Fig. 2a and b). He was described by his parents as a very quiet infant who rarely crying even when receiving vaccinations. He seemed to develop normally, make eye contact, and interact spontaneously until approximately 5-months of age as he no longer made good eye contact afterward. He had gastrointestinal discomfort. The main clinical manifestation was constipation (one stool/3–4 days), with dry knot hard to discharge, and often accompanied by anal fissure. Also he showed a gastroesophageal reflux and a halitosis in the morning. His symptoms relieved after improvement of dietary habits before sleeping, stop the night milk, and improve sleep posture. He had an initial developmental evaluation at 6-months old with a subsequent follow-up. There were concerns about his delayed motor development. He exhibited developmental delays, sitting at 10-months and walking after 18-months of age. He was irritable and cried constantly. He had abnormal social interactions with poor eye contact and stereotypic behaviors. His hearing evaluation at 23-months was normal. By 18-months of age, he had not developed speech appropriately. There were no reports of clinical seizures in this patient. There was no similar disease in the other member of the family.

The patient was assessed by using Bayley scales at 30 months old and the cognitive score was < 50 (22–23 months) and the motor score was 55 (19 months).

The scores of PDMS-2: balance ability (20 months), locomotion (17 months), grasping (23 months), V-M integration (21 months), and standardized motor quotients: GMQ (53), FMQ (76), TMQ (59).

ADOS [18] was used to assess communication, social interaction abilities, creativity and the imaginative use of objects (Table 1). In the “communication” category, it was noted that he did not respond to his name as the patient 1. His verbal and non-verbal communication capabilities were so weak that, at that age, it was clearly observed that the quality of his eye contact, as well as social interactions, were in the autistic spectrum range. He did also have repetitive behaviors. His socialization skills were variable: did not interact with children in his same age and was unable to appreciate social cues. His ADOS scores (Table 1) suggested that the child was in the range of the autistic spectrum. This finding confirmed our primary developmental diagnosis of ASD.

MRI showed the increased signal in T2 in the white matter territories adjacent to the lateral ventricles and subcortical zone, and retardation of brain development (Fig. 2c). EEG showed non specific slow background activity, as well as no epileptiform discharges.

Genetic analyses were performed after obtaining the patient’s signed informed consent and approved by our hospital ethical committee. Exome sequencing revealed a novel heterozygous missense mutations, c.1677C > A (p.M559I) in *CHD8* gene which was further validated by Sanger sequencing (Fig. 2. E1). The variant (p.M559I) was determined to be pathogenic which was classified basing on American College of Medical Genetics and Genomics guideline.

## Discussion and conclusions

In the present study, we report de novo heterozygous missense/nonsense mutations of the *CHD8* gene in two children with autism and global developmental delay that has not been previously reported. This provides additional evidence that disruption of the *CHD8* gene could result in the development of ASD. Increasing evidences from exome sequencing to targeted analysis have showed that de novo loss-of-function mutations in the *CHD8* gene are associated with ASD [7–10, 17, 19]. A decrease in functional *CHD8* in human neural progenitor cells may be another cause of the development of ASD as it induced transcriptional alterations [20]. There are lots of de novo loss of function mutations have been found contribute to ASD risk [14–16]. Many of these genes appear to be involved in regulation of transcription and modification of chromatin [21]. *CHD8* binds to β-catenin in its function in chromatin remodeling [22] and as a potential regulator of Wnt signaling which plays an important role in development [23]. *CHD8* also interacts with other ASD risk genes in neurodevelopment [24]. All these suggest that *CHD8* regulates co-expressing genes during human brain development and most of these genes are associated with ASD. Therefore, *CHD8* mutations could result in intellectual disability and developmental delay as a behavioral profile consistent with ASD, together with macrocephaly with rapid postnatal growth, increased incidences of gastrointestinal problems and sleep disturbance [17]. We performed review of all other 16 cases of *CHD8* nonsense/missense mutations reported in the Human Gene Mutation Database (HGMD) [8, 17, 19, 25–29] (Table 2). Notably, our two *CHD8* nonsense/missence mutation case (c.2647C > A and c.1677C > A) has not been previously reported. *CHD8* mutations are associated with mild intellectual disability, early motor deficits, and speech delay, without distinct structural or EEG brain anomalies. Our patients have these common phenotypic features. In conclusion, we describe two cases of a novel heterozygous missense/nonsense mutations of *CHD8* gene in two Chinese children with autism and global developmental delay, along with other previously reported studies support that disruption of the *CHD8* gene represents a specific genetic sub-type of ASD.

**Table 2:** Comparison of two patient’s findings to 16 other cases of *CHD8* heterozygous missense /nonsense mutations that have been reported in the Human Gene Mutation Database

Features	Patient 1	Patient 2	1[8]	2[19]	3[19]	4[25]	5[17]	6[17]	7[17]
Sex	M	M	M	M	M	M	M	M	M
Age (months)	24	28	96	48	60	216	72	N/A	84
Mutation	Nonsense	Missense	Nonsense	Nonsense	Nonsense	Nonsense	Nonsense	Missense	Missense
Nucleotide/Protein	c.2647C > A/p.E883X	c.1677C > A/p.M559I	c.3712C > T/p.Q1238	c.185C > G/p.S62	c.4009C > T/p.R1337	c.6518C > A/p.S2173	c.2729G > A/p.R910Q	c.3340G > T/p.E1114	c.5129G > T/p.G1710 V
Inheritance	dn	dn	dn	dn	N/A	N/A	dn	N/A	Inherited Maternal
Developmental delay	+	+	+	+	+	–	+	N/A	+
Intellectual disability	+	+	+	–	–	–	+	N/A	N/A
Verbal IQ	N/A	N/A	20	75	79	N/A	27	N/A	N/A
Non-verbal IQ	N/A	N/A	34	78	92	N/A	41	N/A	N/A
Autism	+	+	+	+	+	Schizophrenia	+	Intellectual disability	+
Macrocephaly	+	–	+	–	+	N/A	+	N/A	–
Gastrointestinal	+	+	+	+	+	–	+	N/A	–
			Constipation Abdominal pain	Chronic constipationand loose stool	Constipatio Diarrhea		Constipatio Diarrhea		
Features	8[17]	9[26]	10[27]	11[28]	12[29]	13[29]	14[29]	15[29]	16[29]
Sex	M	M	M	M	N/A	N/A	N/A	N/A	N/A
Age (months)	204	36	264	96	N/A	N/A	N/A	N/A	N/A
Mutation	Missense	Nonsense	Missense	Missense	Missense	Missense	Missense	Missense	Nonsense
Nucleotide/Protein	c.5390G > A/p.R1797Q	c.5500C > T/p.R1834	c.2230G > A/p.V744I	c.3979G > A/p.E1327K	c.856C > T/p.R286C	c.6472C > T/p.R2158C	c.6538C > T/p.R2180C	c.6830G > C/p.G2277A	c.6941G > A/p.R2314Q
Inheritance	Inherited Patenal	dn	dn	dn	N/A	N/A	N/A	N/A	N/A
Developmental delay	N/A	N/A	N/A	N/A	N/A	N/A	N/A	N/A	N/A
Intellectual disability	_	+	+	+	N/A	N/A	N/A	N/A	N/A
Verbal IQ	76	N/A	N/A	N/A	N/A	N/A	N/A	N/A	N/A
Non-verbal IQ	95	N/A	N/A	N/A	N/A	N/A	N/A	N/A	N/A
Autism	+	+	+	–	+	+	+	+	+
Macrocephaly	–	N/A	N/A	N/A	N/A	N/A	N/A	N/A	N/A
Gastrointestinal	+	N/A	N/A	N/A	N/A	N/A	N/A	N/A	N/A
	Diarrhea Constipation								

*Abbreviations*: *dn* de novo, *N/A* data was not available

## Abbreviations

ADOS: Autism diagnostic observation schedule
ASD: Autism spectrum disorders
*CHD8*: Chromodomain helicase DNA-binding protein 8
DSM5: Diagnostic and Statistical Manual for Mental Disorders 5th Edition
FMQ: Fine motor quotient
GMQ: Gross motor quotient
HGMD: Human gene mutation database
NGS: Next-generation sequencing
PDMS-2: Peabody developmental motor scales 2nd edition
TMQ: Total motor quotient
V-M: Visual-motor integration
